# Synthesis of human amyloid restricted to liver results in an Alzheimer disease–like neurodegenerative phenotype

**DOI:** 10.1371/journal.pbio.3001358

**Published:** 2021-09-14

**Authors:** Virginie Lam, Ryusuke Takechi, Mark J. Hackett, Roslyn Francis, Michael Bynevelt, Liesl M. Celliers, Michael Nesbit, Somayra Mamsa, Frank Arfuso, Sukanya Das, Frank Koentgen, Maree Hagan, Lincoln Codd, Kirsty Richardson, Brenton O’Mara, Rainer K. Scharli, Laurence Morandeau, Jonathan Gauntlett, Christopher Leatherday, Jan Boucek, John C. L. Mamo

**Affiliations:** 1 Curtin Health Innovation Research Institute, Faculty of Health Sciences, Curtin University, Bentley, Australia; 2 School of Population Health, Faculty of Health Sciences, Curtin University, Bentley, Australia; 3 School of Molecular and Life Sciences, Faculty of Science and Engineering, Curtin University, Bentley, Australia; 4 Department of Nuclear Medicine, Sir Charles Gairdner Hospital, Nedlands, Australia; 5 School of Medicine and Pharmacology, University of Western Australia, Crawley, Australia; 6 Sir Charles Gairdner Hospital, Nedlands, Australia; 7 Department of Molecular Imaging and Therapy Service, Fiona Stanley Hospital, Murdoch, Australia; 8 Ozgene Pty Ltd, Bentley, Western Australia, Australia; 9 Department of Radiology, Sir Charles Gairdner Hospital, Nedlands, Australia; 10 Laboratory for Cancer Medicine, Harry Perkins Institute of Medical Research, University of Western Australia, Centre for Medical Research, QEII Medical Centre, Australia; 11 Radiopharmaceutical Production and Development Centre (RAPID) PET Laboratories, Sir Charles Gairdner Hospital, Nedlands, Australia; 12 Physics, University of Western Australia, Nedlands, Australia; 13 Health Technology Management Unit, East Metropolitan Health Service, Perth, Australia; Stanford University School of Medicine, UNITED STATES

## Abstract

Several lines of study suggest that peripheral metabolism of amyloid beta (Aß) is associated with risk for Alzheimer disease (AD). In blood, greater than 90% of Aß is complexed as an apolipoprotein, raising the possibility of a lipoprotein-mediated axis for AD risk. In this study, we report that genetic modification of C57BL/6J mice engineered to synthesise human Aß only in liver (hepatocyte-specific human amyloid (HSHA) strain) has marked neurodegeneration concomitant with capillary dysfunction, parenchymal extravasation of lipoprotein-Aß, and neurovascular inflammation. Moreover, the HSHA mice showed impaired performance in the passive avoidance test, suggesting impairment in hippocampal-dependent learning. Transmission electron microscopy shows marked neurovascular disruption in HSHA mice. This study provides causal evidence of a lipoprotein-Aß /capillary axis for onset and progression of a neurodegenerative process.

## Abstract 2

It has been suggested that peripheral metabolism of amyloid-beta is associated with risk for Alzheimer’s disease. This study reveals that the expression of human amyloid exclusively in the liver induces Alzheimer’s disease-like pathologies in mice, potentially indicating a completely novel pathway of Alzheimer’s disease aetiology and therapies.

## Introduction

Several recent bio-epidemiological studies show that systemic measures of amyloid beta (Aβ) in blood positively correlate with cerebral amyloid burden and cognitive decline in Alzheimer disease (AD) [1]. A causal association is suggested based on the findings that blood measures of Aβ isoforms discriminate with a high degree of accuracy, patients who go on to develop AD decades before onset of disease [2]. However, presently, the mechanism(s) by which peripheral Aβ metabolism might exacerbate AD risk are not well understood.

Insight into how blood Aβ increases risk for AD comes from findings that in humans, greater than 90% of blood Aβ_1–40_ and 97% of the particularly pro-amyloidogenic Aβ_1–42_ is associated with plasma lipoproteins [3], principally the triglyceride-rich lipoproteins (TRLs) of hepatically derived very low-density lipoproteins (VLDLs) and of postprandial chylomicrons [4,5]. Direct evidence of a peripheral TRL-Aβ/vascular risk pathway for AD comes from studies in preclinical models, which show that cerebral capillary amyloid-angiopathy, a common early neurovascular pathology of AD, may be a consequence of parenchymal extravasation of TRL-Aβ. In wild-type (WT) C57BL/6J mice, a saturated fatty acid (SFA)-enriched diet was found to strongly stimulate biosynthesis and secretion of TRL-Aβ, concomitant with a reduction in cerebral capillary endothelial tight junction proteins, blood-to-brain extravasation of TRL-Aβ, and marked neurovascular inflammation [6]. In contrast, mice fed unsaturated fatty acid–rich diets had no evidence of exaggerated TRL-Aβ secretion and capillary integrity was unremarkable [6,7]. Studies in transgenic-human amyloid mice also support a causal association with aberrant metabolism of TRL-Aβ. Burgess and colleagues reported that, in TgCRND8 mice, the onset and progression of cerebral amyloidosis was strongly associated with secretion into blood of VLDL-Aβ [8]. Moreover, synergistic effects of TRL-Aβ with exaggerated central nervous system (CNS) synthesis of human Aβ are also suggested by the findings of accelerated amyloidosis in amyloid precursor protein/presenilin 1 (APP/PS1) mice maintained on atherogenic diets [9]. Collectively, it is a reasonable proposition that putative extracellular parenchymal retention of TRL-Aβ is likely to be inflammatory and could promote AD onset or progression. Consistent with the latter, Matsuzaki and colleagues reported that lipidated-Aβ was found to be toxic to Chinese hamster ovary cells in culture, but not native Aβ [10], and in cell cultures studies, macrophages were found to have unabated uptake of TRL [11]. The latter suggests that metabolism of the lipoprotein-Aβ moiety within the neurovascular unit (NVU) might exacerbate inflammatory processes that compromise neurovascular integrity.

The strains of human-amyloid transgenic mice presently available have significant CNS expression of genes that leads to genesis of human Aβ, of more toxic isoforms Aβ, or in combination with other proteins of interest such as tau. While providing valuable insight into understanding pathological sequelae in AD, the CNS dominant expression of genes in these models is a significant confounder to explore the hypothesis of whether the peripheral metabolism of TRL-Aβ is causally associated with AD as a consequence of TRL-Aβ capillary dysfunction and parenchymal extravasation. To address this limitation, for this study, we developed a new murine transgenic model that was modelled on the widely used APP1 knock-in model expressing human *APP 695* isoform containing the Swedish (KM670/671NL) and Indiana (V717F) transgenes. We engineered mice with expression of the said genes restricted to liver hepatocytes (the hepatocyte-specific human amyloid (HSHA) strain). Developed using a ROSA-cre conditional knock-in approach, the HSHA mice have no expression of human-Aβ in brain, nor in other peripheral nonlipogenic peripheral organs. The HSHA strain, therefore, represents a unique opportunity to specifically explore the TRL-Aβ lipoprotein metabolic cascade in the context of neurovascular integrity and AD risk.

## Results

### Human APP gene expression is restricted specifically to the liver of HSHA mice

**Fig 1:**
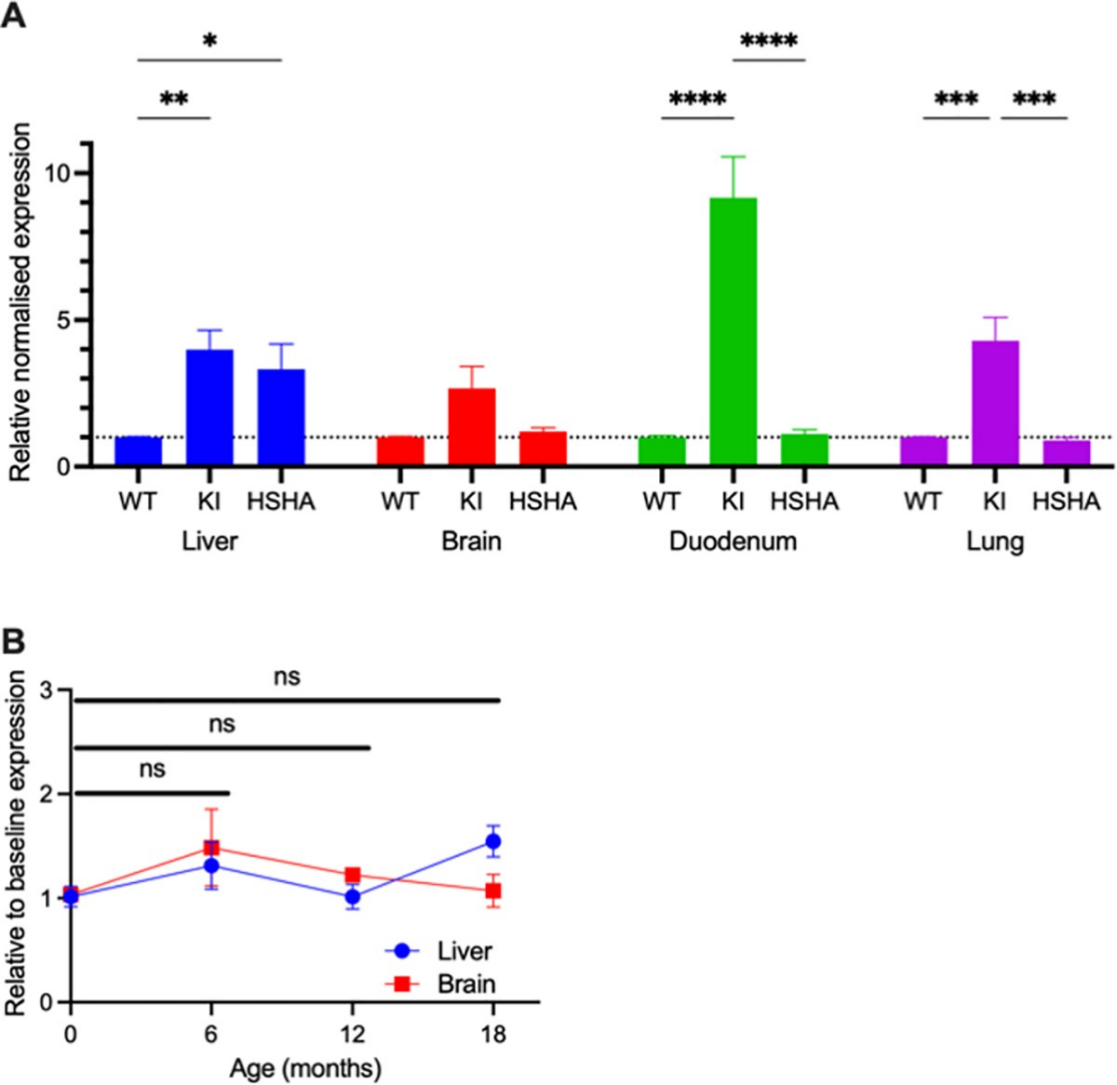
mRNA expression of human APP relative to reported gene Eef2. The APP qPCR assay is designed to only detect human APP using a locked nucleic acid approach. The assay was tested against WT cDNA isolated from WT mice, and no amplification was observed, indicating that the assay was specific to human APP sequence only. (A) Detection of qPCR signal demonstrates the expression of Human *APP* in mRNA. qPCR of RT-cDNA was performed in duplex with Eef2 (MGI: 95288) as an endogenous control. Expression of the read-through is arbitrarily set a value of 1, and then the relative expression level of APP following breeding to cre, either liver-specific Alb-cre (HSHA) or germline OzCre deletor (KI), is compared to it. Data are presented as mean ± SEM relative normalised expression (*n =* 3). Two-way ANOVA followed by Fisher LSD post hoc multiple comparison was used to determine the statistical significance (* *p* < 0.05, ** *p* < 0.01, *** *p* < 0.001, **** *p* < 0.0001). (B) Human APP mRNA expression was also measured with qPCR in liver and brain tissues at 6, 12, and 18 months of age. Data are presented as mean ± SEM expression relative to baseline. Two-way ANOVA followed by Fisher LSD post hoc multiple comparison was used to determine the statistical significance, and no significance was detected in both brain and liver expression of human APP mRNA. The data underlying Fig 1 can be found in S1 Data. Eef2, eukaryote translation elongation factor 2; HSHA, hepatocyte-specific human amyloid; ns, not significant; WT, wild-type.

Fig 1 depicts the median mRNA human *APP* gene expression relative to the reporter gene, Eef2, in the tissue hepatic conditional knock-in HSHA (*hu_APP1*^*lox-STOP-lox*^*+Alb-cre*) strain and the full-body knock-in (KI) strain (*hu_APP1*^*lox-STOP-lox*^*+OZ-cre*) unconditional transgenic strain (ROSAKI). As required for this study, the HSHA mice have significantly higher expression in liver, but not in brain (Fig 1A). In contrast, the unconditional ROSAKI strain showed expression in a range of tissues including the brain, lung, and liver. We assessed expression of human APP mRNA at 6, 12, and 18 months of age and confirm no significant difference compared to the baseline (Fig 1B).

The mice did not show any clinical adverse effects, while the HSHA mice had significantly greater weight gain at 12 and 18 months of age (S1 Fig).

### Hepatic-hAPP induced an age-associated increased cerebral uptake of amyloid radiotracer

**Fig 2:**
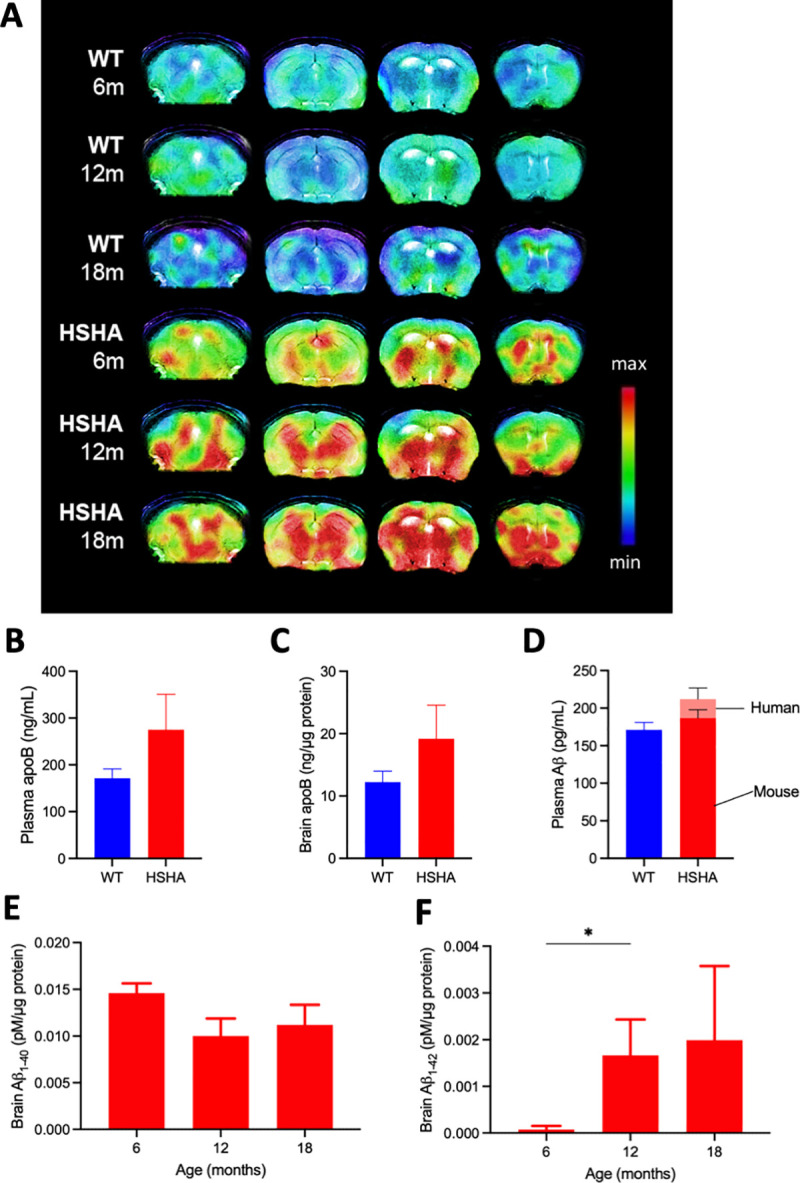
Plasma and brain concentrations of apo B and Aβ. (A) In vivo PET detection of amyloid binding was used to confirm cerebral amyloid deposition in HSHA mice and their age-matched WT controls. Representative PET and MRI fusion images (coregistered with MRI templates using conventional Fast Spin Echo Sequence T2-weighted sequence) show scan data at 20 to 40 minutes’ post-intravenous administration of [^11^C] PiB. Each panel (from left to right) illustrates maximum binding potential for [^11^C] PiB represented by coronal images at −2, 0, 4, and 6 mm from the bregma; indicated by the vertical bar. Plasma (B) and brain (C) levels of apo B, a surrogate marker of TRLs in HSHA and WT control mice, were determined with ELISA. Data are expressed as mean ± SEM. Human and mouse isoforms of Aβ in plasma (D) and brain (E, F) were measured separately with ELISA kits using antibodies specific to each isoform. The data underlying Fig 2B–2F can be found in S1 Data. Aβ, amyloid beta; apo B, apolipoprotein B; HSHA, hepatocyte-specific human amyloid; MRI, magnetic resonance imaging; PET, positron emission tomography; PiB, Pittsburgh compound; TRL, triglyceride-rich lipoprotein; WT, wild-type.

In addition to the in vitro analyses of human Aβ abundance in the brain of HSHA mice as above, the global abundance of Aβ in HSHA and control mice was studied in vivo by amyloid-binding tracer, [^11^C] Pittsburgh compound (PiB), positron emission tomography (PET) imaging, coregistered to magnetic resonance imaging (MRI)-acquired data for structural annotation. Fig 2A depicts the distributional uptake of [^11^C]-PiB in control and in HSHA mice with increasing age. The SUVR_WB:CBL_, which describes the standardised uptake value ratios of whole brain to cerebellum, is also provided in Table 1. We found an SUVR for whole brain relative to cerebellum of 0.92 ± 0.02 for C57BL/6J mice 6 to 18 months of age. In the HSHA mice with the Swedish mutation expressed in liver, the SUVR_WB:CBL_ was 0.97 ± 0.01 at 6 months of age, 1.00 ± 0.02 at 12 months of age, and 1.08 ± 0.06 at 18 months of age.

With data capture collected and analysed blind to strain and age, the results show that PiB signal intensity was not realised in WT mice irrespective of age. In contrast, HSHA mice showed significant signal intensity for PiB-PET, including within cortex (CTX), within the hippocampal formation (HPF), and within the thalamus. The PiB signal intensity was clearly positively associated with increasing age in HSHA mice.

**Table 1:** SUVR values of ^11^C PiB in WT and HSHA mice.

Strain	Age (months)	PiB-PET SUVR_WHOLE BRAIN: CEREBELLUM_
HSHA	6	0.97 ± 0.01
	12	1.00 ± 0.02
	18	1.08 ± 0.06*
WT (C57BL/6J)	6–18	0.92 ± 0.02

Values are mean ± SEM, HSHA: *n =* 3/group, WT: *N =* 9; * denotes *p* < 0.05 vs WT controls. The data underlying Table 1 can be found in S1 Data.

SUVR, standardised uptake value ratio; PET, positron emission tomography; PiB, Pittsburgh compound; WT, wild-type.

### Liver-specific hAPP increased plasma and brain TRL-hAβ

Compared to WT mice, at 6 months of age, we found that HSHA mice have approximately a 40% greater abundance in blood of the apolipoprotein (apo) B lipoproteins, which chaperone Aβ when secreted from lipogenic organs (Fig 2B). The finding is consistent with the hypothesis of exaggerated vascular exposure to TRL-Aβ. Moreover, brain abundance of apo B also increased in HSHA mice compared to controls (Fig 2C).

Concomitant with the increase in plasma apo B, we show that the total abundance of Aβ in blood was increased by approximately 30% in HSHA mice compared to WT controls (Fig 2D). We show that the greater concentration of Aβ in HSHA mice was a consequence specifically of increased abundance of the human Aβ isoform. The concentration of murine Aβ in HSHA mice remained comparable to WT controls. Furthermore, in brain, we confirm the abundance of human Aβ isoform, indicating the blood-to-brain kinetics of TRL-Aβ (Fig 2E and 2F). We observed a significant increase of human Aβ_1–42_ in the brain tissue of HSHA mice from 6 to 12 months of the age.

### Hepatic APP expression in HSHA mice causes aberrant AD-like accumulation of neutral lipids in the brain

**Fig 3:**
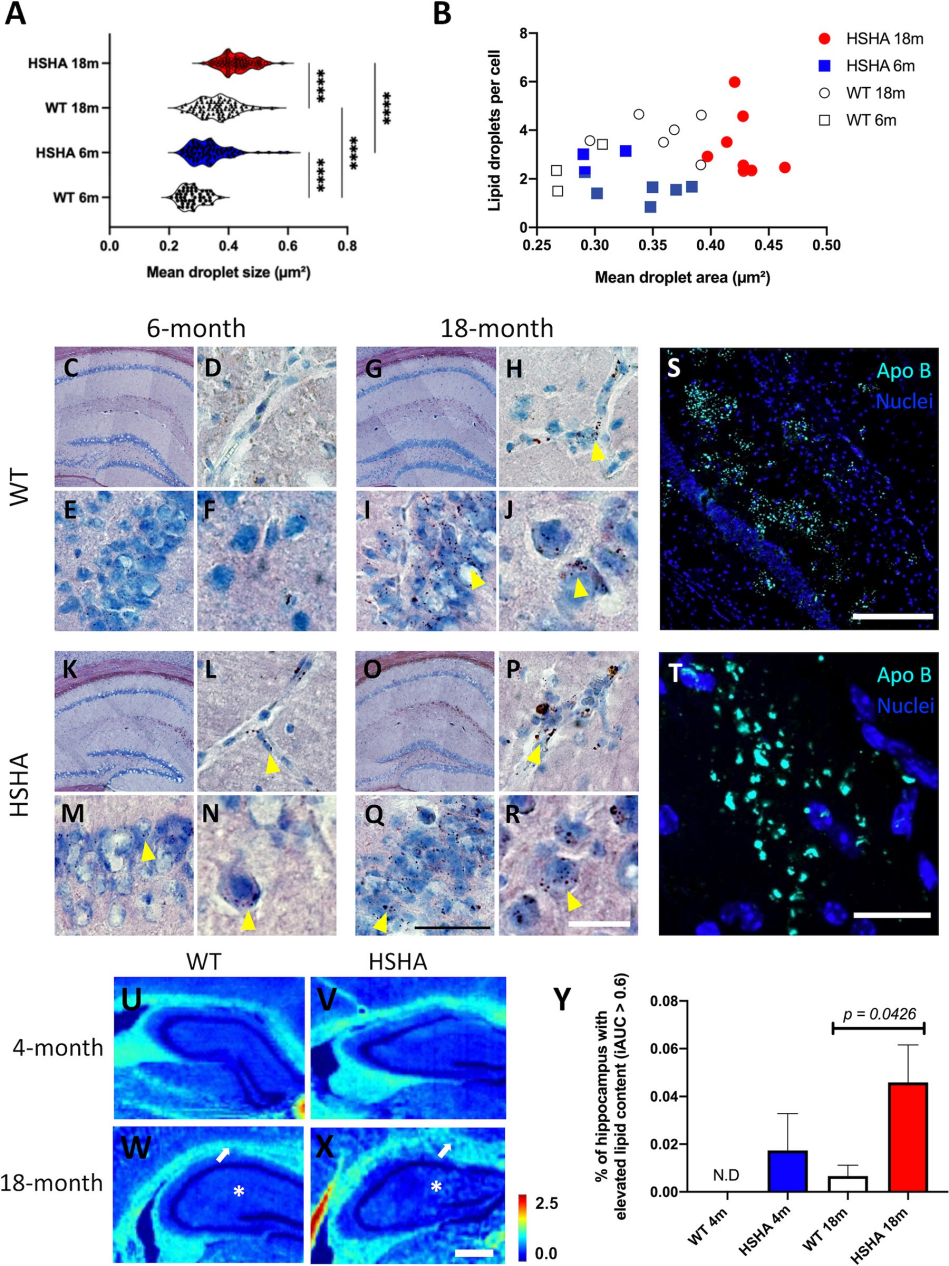
Lipid droplets are present in HSHA brain HPF at both 6 and 18 months of age. **(A)** The distribution of the average size of lipid droplets stained by Sudan IV lipid stain in the HPF of 6-month-old (blue) and 18-month-old (red) HSHA mice with their age-matched controls. Data are expressed as means; *n =* 4–8 mice with approximately 100 images analysed. Statistical significance was assessed with two-way ANOVA (**** *p* < 0.0001). **(B)** The Pearson correlation coefficient of the mean area occupied by lipid droplets and number of lipid droplets per cell in the HPF region in young and aged HSHA and WT controls (*n* = 4–8). **(C, G, K, O)** Representative bright field coronal micrographs of the HPF stained with Sudan IV in 6-month-old and 18-month-old HSHA mice and their age-matched WT controls. **(D, H, L, P)** Higher magnification of the HPF indicates a greater abundance/size of lipid droplets in blood vessels of the HPF of 18-month-old HSHA mice when compared to younger HSHA mice and their age-matched controls; indicated by the yellow arrowheads. **(E, I, M, Q)** Sudan IV positive-stained cells in the hippocampal CA1 pyramidal neuronal layer are indicated in higher abundance and size in aged HSHA mice compared to younger HSHA mice and their respective controls. **(F, J, N, R)** An abundance of interneuronal lipid droplets in the hippocampal CA1 stratum radiatum and pyramidal layers can be seen in both strains of aged mice; however, the 18-month HSHA cohort of mice (R) exhibited larger lipid droplets when compared to (J). Yellow arrowheads indicate lipid droplets stained by Sudan IV; black scale bars represent 50 μm (D, E, H, I, L, M, P, Q); white scale bars 20 μm (F, J, N, R). **(S)** Representative immunomicrograph of apo B staining (cyan) in the HPF of 12-month-old HSHA mice; scale bar represents 200 μm. **(T)** Representative magnified immunomicrograph of apo B staining (cyan) in the HPF of 12-month-old HSHA mice; scale bar represents 20 μm. **(U-X)** FTIR spectroscopy analysis of hippocampal lipid accumulation in HSHA mice. Representative images of lipid abundance in 4- and 18-month-old WT controls **(U, W)** and HSHA **(V, X)** mice are indicated; white arrow depicts regions of greater lipid abundance (more light blue pixels) within the HPF in aged HSHA mice; scale bars represent 500 μm **(Y).** Quantitative abundance of lipid in the HPF of HSHA mice and their age-matched controls at 4 and 18 months of age. Data are presented as mean ± SEM (*n =* 5; two-way ANOVA; *p*-values only indicated for significance). The data underlying this figure can be found in S1 Data. apo B, apolipoprotein B; HPF, hippocampal formation; HSHA, hepatocyte-specific human amyloid; iAUC, integrated area under the curve; N.D., not detected; WT, wild-type.

Brain parenchymal pro-inflammatory lipid inclusion bodies (LIBs) of neutral lipids (triglyceride and cholesteryl esters) have been reported to increase naturally with ageing but are of unknown aetiology. Given the hypothesis proposal of exaggerated lipoprotein-Aβ penetrance, an established quantitative immunohistochemical (IHC) analysis was adopted to study age-associated LIBs in HSHA mice. Utilising Herxheimer (Sudan IV) neutral lipid staining and with abundance of lipid accumulation analysed blind to strain and age, this study found that HSHA mice had significantly accelerated onset and progression of LIBs within the HPF and CTX compared to age-matched control mice (Fig 3A and 3B). Fig 3 also demonstrates Herxheimer LIBs within the CA1 pyramidal neuronal layer, and higher magnification showed a focal propensity for LIBs within and adjacent to blood vessels of the HPF (depicted in frames D, H, L, and P). Extending on the quantitative IHC Herxheimer staining described, Fourier transform infrared (FTIR) spectroscopy of the HPF equivocally confirmed a marked age-associated accumulation of neutral lipids (triglyceride/esterified cholesterol) throughout the HPF in HSHA mice compared to aged-matched WT controls (Fig 3Y). To explore if the accelerated accumulation of LIBs/neutral lipid demonstrated by Herxheimer staining and FTIR might potentially reflect exaggerated extravasation of blood-derived TRLs, we determined parenchymal distribution of apo B, an exclusive marker of TRL and their remnants. Apo B is an obligatory large molecular weight structural protein that remains with the lipoprotein moiety throughout the catabolic cascade. Fig 3S provides an example of significant substantial clustered abundance of apo B within the HPF of 12-month-old HSHA mice.

### HSHA mice exhibited a marked neurodegenerative phenotype and brain atrophy

**Fig 4:**
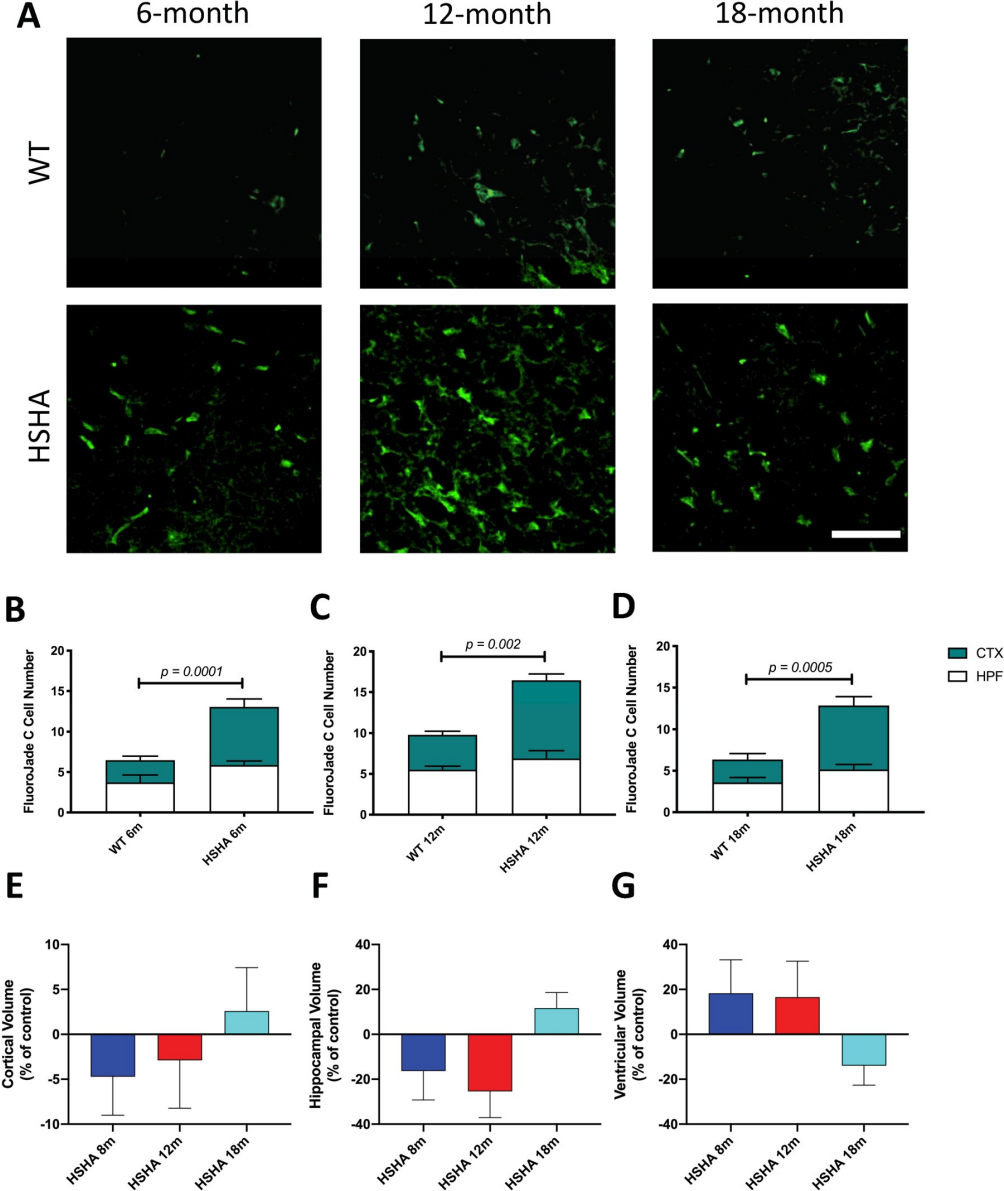
Neuronal degeneration and brain volumetric analyses in HSHA mice. **(A)** Representative 3D confocal immunomicroscopy images of degenerated neurons in HSHA mice and their respective controls at 6, 12, and 18 months of age, indicated by FluoroJade C staining in green; scale bar depicts 50 μm **(B–D) Quantitative analysis of neuronal degeneration in HSHA mice.** The number of degenerative neurons, as indicated by FluoroJade C positive cells assessed by quantitative confocal immunomicroscopy, is shown in 6-month-old **(B**), 12-month-old **(C),** and 18-month-old **(D)** HSHA mice and their respective age-matched controls, in the CTX and HPF. Statistical significance was tested by an unpaired *t* test with Welch correction testing for nonequivalence of standard deviations. Data are presented as mean ± SEM (*n =* 4–10; *p*-values only indicated for significance). **(E–G) Comparison of regional brain volumes measured by in vivo 3D T2-weighted MRI in young and old HSHA mice.** The percentage volume difference between **(E)** CTX, **(F)** HPF, and **(G)** combined lateral, third, fourth, and cerebral aqueduct VNT volumes versus the respective regional mean volume of HSHA mice and their WT controls at 8, 12, and 18 months, are indicated. Each image data set was reconstructed, processed, coregistered with the Allen Mouse Brain Atlas, and analysed per region of interest (*n =* 2–3). Treatment differences were assessed by one-way ANOVA by comparing the percentage volume difference for HPF, CTX, and VNT size versus the respective regional mean volumes at 8, 12, and 18 months of age. No significant differences were observed at *p* < 0.05. The data underlying this figure can be found in S1 Data. CTX, cortex; HPF, hippocampal formation; HSHA, hepatocyte-specific human amyloid; MRI, magnetic resonance imaging; VNT, ventricular; WT, wild-type.

To explore if HSHA mice were developing an accelerated neurodegenerative phenotype concomitant with accelerated lipid/lipoprotein parenchymal retention, Fluoro Jade C stain, a marker of degenerated neurons, was utilised. With data collected blind to treatment, analysis revealed that across the life span of HSHA mice to 18 months of age, neurodegeneration was approximately 2-fold greater compared to age-matched control mice (Fig 4B–4D). Furthermore, a TUNEL assay revealed in 12 and 18 months old HSHA mice that approximately 4-fold increase in the number of apoptotic cells compared to age matched WT control (S2 Fig). The chronic degenerative and apoptotic phenotype in HSHA was supported by brain volumetric analysis. Utilising MRI, HSHA mice showed a 34% reduction in HPF volume at 12 months of age compared to controls and a 14% reduction in size for CTX (Fig 4E and 4F and Table 2). The changes in HPF and CTX were comparable to clinical reports in patients transitioning from mild cognitive impairment to established AD [12,13]. At 18 months of age, the HSHA then showed evidence of global swelling particularly within the cerebral CTX, a phenomenon reported in other established amyloid transgenic strains such as APP/PS1 mice [14]. Ventricular size was inversely associated with the regional changes in brain volume, with larger ventricles indicated at 8 and 12 months of age in HSHA mice compared to controls, and a subsequent reduction in oedema was realised (Fig 4G and Table 2).

**Table 2:** Regional brain volume measurements (mm^3^) and their variation with age in WT and HSHA mice at 8, 12, and 18 months.

Brain regions	8 months		12 months		18 months	
	WT	HSHA	WT	HSHA	WT	HSHA
Cerebral CTX	172.51 ± 6.91	164.36 ± 7.41	158.16 ± 2.35	153.63 ± 8.45	172.95 ± 0.97	177.48 ± 8.36
HPF	32.20 ± 3.82	26.93 ± 4.13	31.63 ± 1.61	23.58 ± 2.68	20.54 ± 0.36	22.94 ± 1.43
Ventricles^a^	20.20 ± 1.49	23.89 ± 3.01	20.15 ± 1.35	23.49 ± 3.22	20.19 ± 0.57	17.38 ± 1.75

Values are mean ± SEM; *n* = 2–3/group. The data underlying Table 2 can be found in S1 Data.

^a^Total ventricle comprises the sum of the lateral, third, and fourth ventricle.

CTX, cortex; HPF, hippocampal formation; WT, wild-type.

### Restriction of human APP to liver hepatocytes exacerbates cerebral capillary dysfunction and neurovascular inflammation

**Fig 5:**
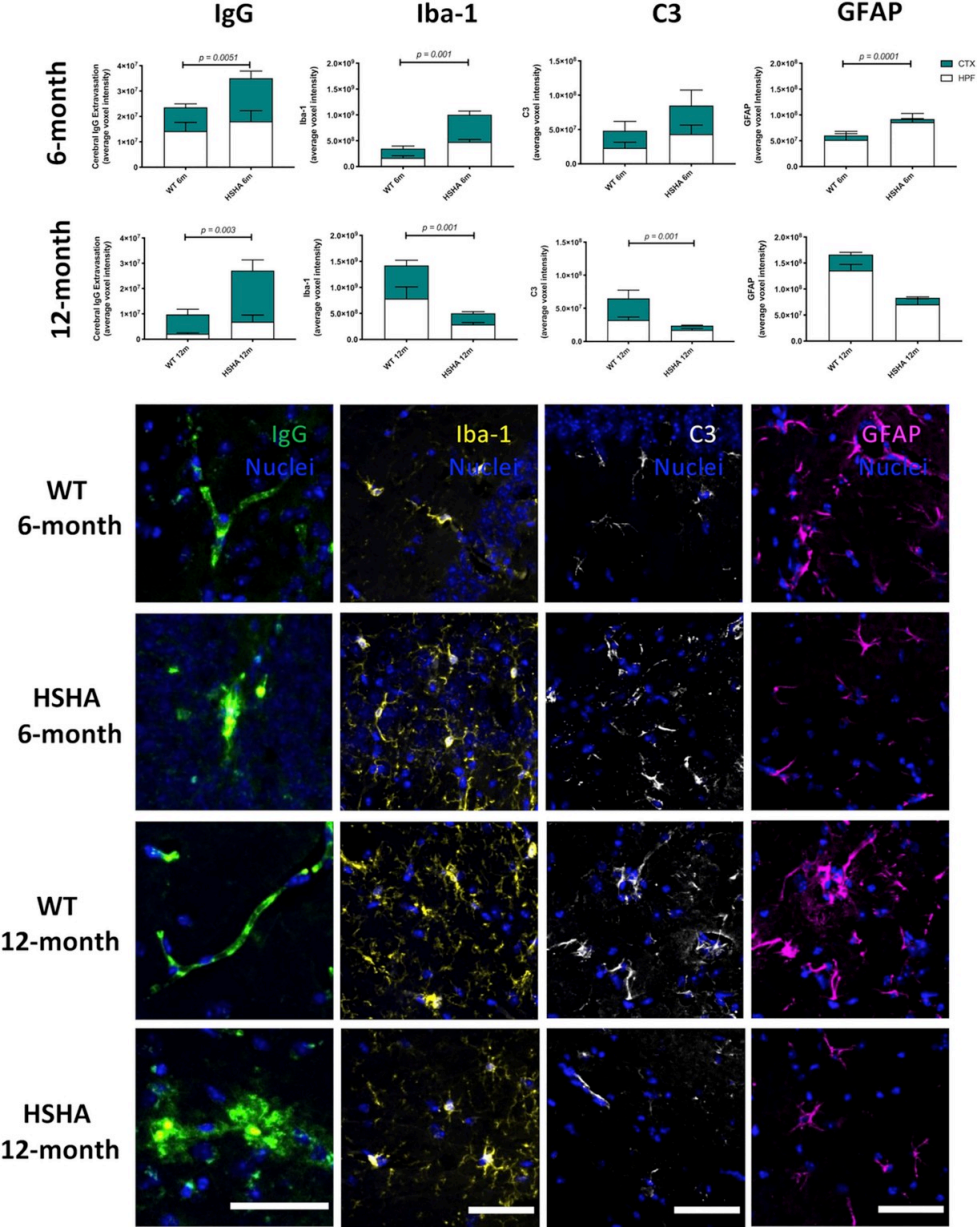
Three-dimensional confocal immunomicroscopy analysis of markers of cerebral capillary integrity, microglial activation, and astrocytic immunoreactivity. Increased cerebral capillary permeability, activated microglia, A1 reactive astrocytes, and activated astrocytes were assessed by the quantitative immunoreactivity of cerebral extravasation of plasma protein IgG, Iba-1, C3, and GFAP, respectively, in the cerebral CTX and HPF of HSHA mice and their age-matched WT controls at 6 **(A–D)** and 12 months **(E–H)** of age. The latter frames, which are representative confocal immunomicrographs of IgG (green), Iba-1 yellow), C3 (white), and GFAP (magenta), are presented (nuclei are shown as blue; scale bar = 50 μm). Statistical significance between each strain and age were tested by an unpaired *t* test with Welch correction testing for nonequivalence of standard deviations. Data are presented as mean ± SEM (*n =* 4–12; *p*-values only indicated for significance). The data underlying Fig 5 A–H can be found in S1 Data. CTX, cortex; C3, complement component 3; GFAP, glial fibrillary acidic protein; HPF, hippocampal formation; HSHA, hepatocyte-specific human amyloid; Iba-1, ionised calcium-binding adaptor molecule 1; IgG, immunoglobulin G; WT, wild-type.

In HSHA mice, we confirm that neurodegeneration was associated with earlier age onset of increased capillary permeability within both the HPF and CTX regions. Fig 5A depicts the parenchymal abundance of immunoglobulin G (IgG), an established marker of blood–brain barrier dysfunction. We also observed significant reduction in the expression of blood–brain barrier tight junction protein, occludin-1, in HSHA mice, compared to age-matched WT controls (S3 Fig). The loss of tight junction colocalized with the parenchymal IgG extravasation in HSHA mouse brain. Our analysis also confirmed that there were no changes in the vascular density in HSHA mice compared to age-matched WT control mice (S4 Fig).

Concomitant with capillary dysfunction, realised by 6 months of age in HSHA mice was the confirmation of a neurovascular inflammatory response with significant strain differences indicated for Iba-1 (Fig 5B) and reactive astrogliosis (Fig 5C). Note, the latter was in the absence of marked differences in reactive astrocytes per se (glial fibrillary acidic protein (GFAP); Fig 5D). However, at 12 months of age and despite persistent exaggerated capillary permeability and neurodegeneration (Fig 5E), strain differences for measures of inflammation were no longer realised (Iba-1 or C3; Fig 5F and 5G, respectively). The absence of treatment effects at a later age possibly reflects a delayed inflammatory response in the control mice, which was markedly increased at 12 months of age compared to 6 months of age. Collectively, in both HSHA and control mice, the immunodetection inflammatory indices of Iba-1 and C3 were found to be a transient phenomenon; however, this appeared with earlier onset in HSHA mice.

### Ultrastructure analysis of the brains of HSHA mice revealed marked cellular and subcellular degenerative changes

**Fig 6:**
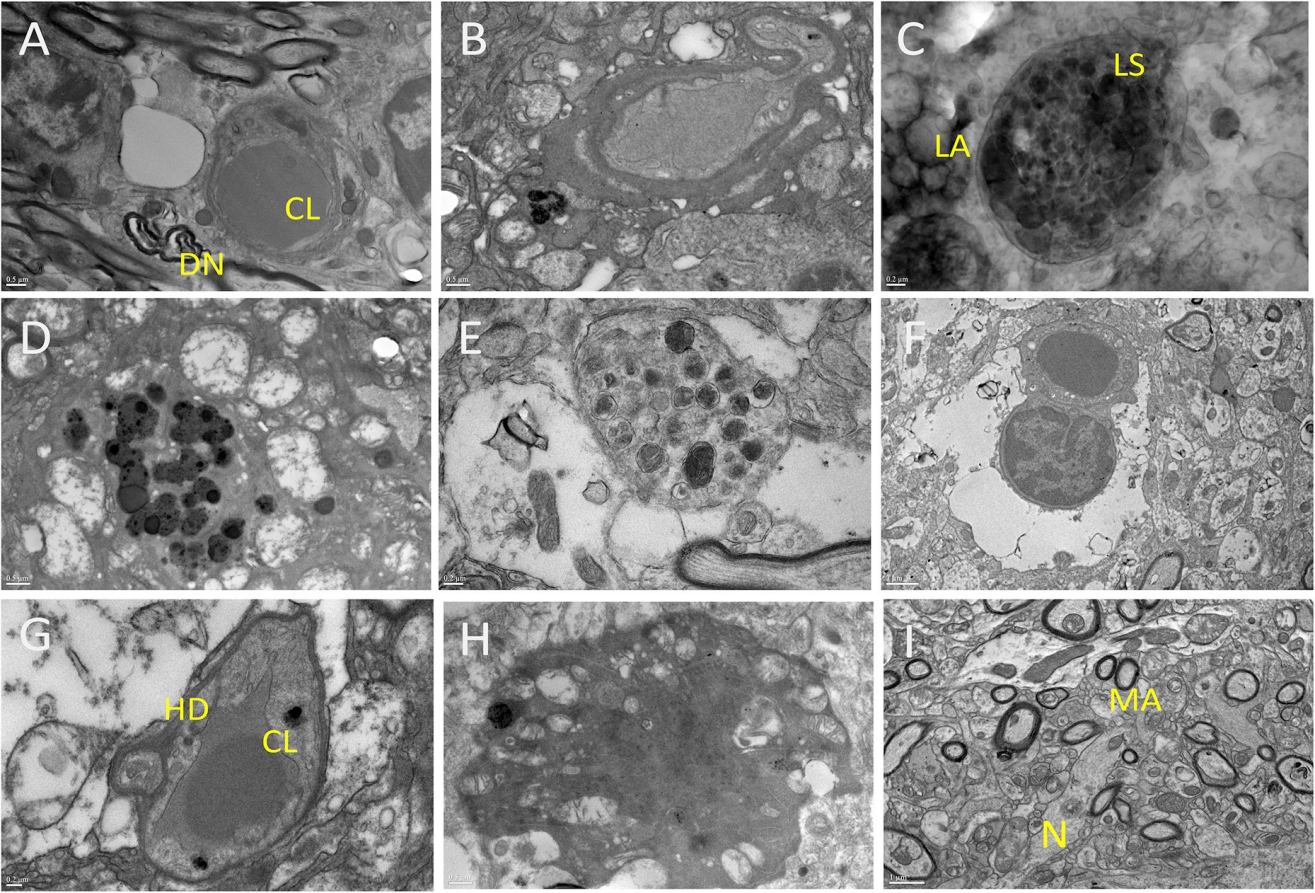
TEM of 12-month-old HSHA hippocampi. **(A)** depicts a CL, with adjacent lipid vesicles, which were common adjacent to DNs; **(B)** shows a distorted capillary, lysosomes adjacent to lipid vesicles and some lipofuscin material; **(C)** shows significant abundance of LS associated with LAs; **(D)** shows a lysosome adjacent to extensive neuronal degeneration; **(E)** shows a dystrophic neurite associated with swollen astrocytes; **(F)** shows extraordinarily large pericyte projection and swollen astrocytic processes adjacent to lipid infiltrated parenchyma with substantial cellular loss; **(G)** show commonly observed residual capillaries surrounded by widespread HD; **(H)** depicts a dark glial cell, widely abundant in HSHA mice but not in aged-matched controls. (**I**) **12-month-old WT hippocampi** normal brain parenchyme including MAs and N. Scale bars represent 0.5 μm for frames A, B, D, H; 0.2 μm for frames C, E, G; 1 μm for frames F and I. CL, capillary vessel; DN, dystrophic neuron; HD, cellular debris; HSHA, hepatocyte-specific human amyloid; LA, lipid aggregate; LS, lysosomal activity; MA, myelinated axon; N, neuron; TEM, transmission electron microscopy; WT, wild-type.

A markedly accelerated neurodegenerative phenotype in HSHA mice was confirmed by transmission electron microscopy (TEM) (Fig 6). The HSHA mice showed significant lipofuscin aggregates developing within cerebral capillaries as early as 8 months of age (Fig 6A–6C). Associated with the significantly affected capillary vessels with compromised lumen were grossly enlarged astrocytic end processes often devoid of intracellular organelles (Fig 6E and 6F). In more severely affected capillaries, there was often substantial abundance of hydrolysed cell debris (Fig 6G). Within brain parenchyma, HSHA mice frequently showed significant large clusters of lipofuscin aggregates, neuronal dystrophy, and mega-large activated dark glial cells (Fig 6H). In contrast, modest lipofuscin material and small dark glial cells were rarely seen in control mice and only at an older age (12- and 18-month C57BL/6J mice).

### HSHA mice showed poorer performance in passive avoidance test

**Fig 7:**
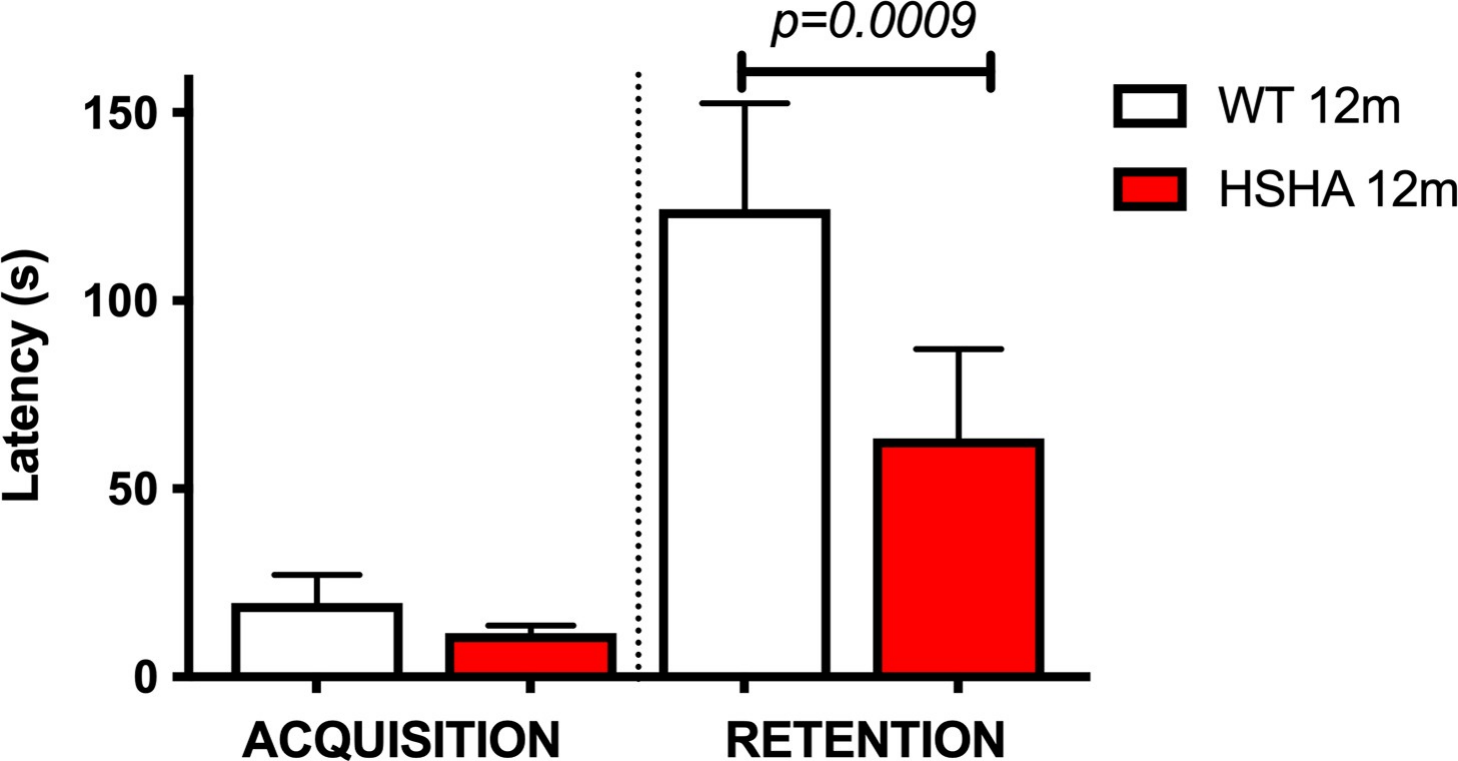
Hippocampal learning behaviour in 12-month-old HSHA mice and their age-matched controls determine by passive avoidance testing. Average latency time in seconds for each group of mice was measured. Statistical significance was assessed by nonparametric Krusal–Wallis test, followed by Dunn multiple comparisons test. Data are presented as mean ± SEM; *n =* 6 for WT and *n* = 15 for HSHA; *p*-values only indicated for significance. The data underlying Fig 7 can be found in S1 Data. HSHA, hepatocyte-specific human amyloid; WT, wild-type.

Hippocampal-dependent learning in HSHA mice was assessed using the widely utilised passive avoidance electric foot shock challenge. The learning or “acquisition” and retention measures indicated impaired hippocampal-dependent learning in HSHA mice. For the primary measure of the retention of learning challenge, the HSHA mice were found to perform approximately half as well as age-matched controls (Fig 7).

### HSHA mice indicated transient modest liver dysfunction

We tested the liver function with alanine aminotransferase (ALT) activity and examined the liver tissue morphology with hematoxylin and eosin staining, in comparison to age-matched WT control (S5 Fig). We found that in HSHA mice, no increase in ALT was observed at 6 months of age compared to the control mice. However, at 12 months of age, HSHA mice showed significant elevation in plasma ALT, which was accompanied by moderate steatosis. Subsequently at 18 months of age, liver function was resolved to comparable level with age-matched WT mice.

## Discussion

Here, we assessed whether an APP-modelled transgenic amyloid strain of mice with expression of human *APP1* restricted to liver hepatocytes (HSHA) develops a neurodegenerative phenotype that could explain aetiology of AD. Our results show that hepatic-only expression of genes leading to the synthesis of human-Aβ results in accelerated evolution of LIBs; parenchymal extravasation of apo B lipoproteins, the primary chaperone of blood Aβ; earlier onset of marked neurovascular inflammation; exaggerated cerebral abundance of Aβ; chronically exaggerated rates of neurodegeneration; brain atrophy in regions associated with cognitive performance; and impaired hippocampal-dependent learning. Compared to commercially available strains of transgenic human-Aβ mice, the HSHA strain to 18 months of age did not show frank parenchymal amyloidosis. Rather, in HSHA mice, we herein show marked abundance of capillaries with lipofuscin aggregates, morphologically aberrant astrocytes and pericytes, and massively enlarged dark glial cells. Our collective findings in HSHA mice of accelerated focal lipocentric CNS aberrations suggest that the peripheral metabolism of TRL-Aβ may be causally associated with a neurodegenerative phenotype. We contend the interpretation is consistent with findings published by Alois Alzheimer decades ago that have been rarely considered in the context of aetiology. Alois Alzheimer described in brain specimens of patients with AD lipofuscin aggregates within small arteries, lipid aggregates adjacent to glial cells and in advanced plaque, and substantial neutral lipid infiltration [15,16].

Preceding the evolution of the HSHA strain, several murine transgenic-amyloid models of AD were developed and widely studied. A common feature is the dominant expression of genes within the CNS, principally modelling familial AD. The first strain of amyloid transgenic mice developed carried the Indiana mutation (V717F) of amyloid precursor protein (APP) [17], and these mice were reported to present with amyloid plaque commencing from just 6 to 9 months of age. The Tg2576 model included the commonly used insertion of the Swedish double mutation (K670N and M671L) [18,19] resulting in the substantial overproduction of total Aβ from the APP. In strains expressing mutations in presenilin (PS) genes alone, these mice were reported to have exaggerated biosynthesis of AB_1-42_ relative to AB_1-40_ and phenotypically manifest with enhanced Aβ aggregation. Unlike APP mice, PS transgenic do not display memory impairments, nor prominent amyloid plaque [20] per se. A widely used combination of APP and PS1 (APP/PS1) coexpress both human transgenes for *APP* (Swedish mutation) and the *PSEN1* (L166P) mutation and exhibit widespread diffuse plaque and significant neuronal loss compared to APP transgenics alone. While the APP and PS transgenic models and, in more recent years, the availability of combination amyloid/tau transgenic mice provide valuable insight of potential pathological sequelae, no particular strain models explain the physiological evolution of sporadic AD particularly well. Rather, in nonfamilial AD, the body of evidence suggests that aetiology is not principally a consequence of CNS overproduction of Aβ per se [21–24].

A large body of epidemiological and, in more recent times, clinical studies suggest that cerebrovascular inflammation is, at the very least, an amplifier of AD progression. Indeed, a recent study by Senatorov and colleagues reported that an anti-inflammatory intervention targeting blood–brain barrier function effectively reinstated neural function and improved cognitive outcomes in aged mice [25]. Previous studies in WT mice and transgenic amyloid mice fed an atherogenic diet suggest that the neurovascular inflammatory trigger/amplifier may reflect chronically exaggerated vascular exposure to lipoprotein-Aβ, which is synthesised and secreted by liver hepatocytes and absorptive epithelial cells of the small intestine, respectively. In order to explore specifically the hypotheses of a peripheral lipogenic organ-derived Aβ/vascular origin for AD, in this study, we generated, for the first time, a new strain of Aβ transgenic mice with expression of human *APP1* (Swedish (KM670/671NL) mutation transgenes restricted exclusively to liver hepatocytes. The HSHA mice provide a differential opportunity to explore neurovascular integrity in the context of peripheral metabolism of human Aβ.

The HSHA mice studied herein showed accelerated progression of age-associated LIBs adjacent to and within blood vessels and within the deeper cortical parenchyma. The findings extend on recent observations in WT mice reported by Shimabukoro and colleagues, who showed numerous multilocular LIBs in the parenchyma and within the perivascular space [26]. In addition, LIBs were seen in the striatum, the primary target of cortical input to the basal ganglia that is mainly involved in motor function, memory, and cognition. Asymmetrical distribution of LIBs was seen in cerebral ventricle walls, which were findings also observed in this study (S6 Fig). Interestingly, Shimabukuro and colleagues reported LIB’s expression of proteins, considered surrogate markers of active autophagolysosomal degradation, concomitant with the production of pro-inflammatory cytokines. They concluded that the LIBs were probably causally associated with a neuroinflammatory process, a proposition that is broadly supported by findings in this study. However, Shimabukuro and colleagues did not consider the origin of LIBs or relevance to AD risk per se. Interestingly, accumulation of neutral lipids within ependymal cells have been reported in both postmortem AD brains and a triple-transgenic murine model of AD (3xTg-AD) [27]. In this study, we extend our understanding of age-associated focal changes in cerebral neutral lipid aggregates by utilising FTIR. Consistent with the Herxheimer staining data showing accelerated LIB formation in HSHA mice, FTIR analysis showed a significantly greater abundance of triglyceride/cholesteryl-esters within the HPF, consistent with findings reported for the association of lipids with amyloid pathology during AD in APP/PS1 mice [28–30] and clinical human tissue [29,31].

Several studies have previously shown parenchymal abundance of apo B in clinical [32] and preclinical studies [33], suggesting that penetrance of the entire lipoprotein macromolecule is a naturally occurring physiological phenomenon, although ordinarily limited by the robust barrier properties of capillary vessels [34]. However, previous studies have demonstrated that when capillary function is compromised by way of vascular insult, for example, via provision of atherogenic diets, markedly increased parenchymal penetrance and retention of TRL-Aβ occurs; neurovascular inflammation ensues and premature development of cognitive deficits are realised. Intervention studies that suppress either lipoprotein-Aβ synthesis and/or neurovascular inflammation confer capillary restorative benefit and attenuate cognitive decline, findings consistent with the hypothesis of TRL-Aβ induced causality [35–37]. This study builds on those findings and now equivocally demonstrates a vascular-axis cascade for AD-like risk in HSHA mice, but indeed without the potentially confounding effects of lipotoxic diets, or substantial CNS expression of genes leading to the synthesis of Aβ.

This study provides, for the first time, direct evidence that hepatic expression of genes leading to the synthesis of human-Aβ is causally associated with corruption of the NVU, with cell death and brain atrophy realised. A paradoxical observation in this study was that classical markers of inflammation (Iba-1 and C3) were transiently realised in HSHA mice, and, indeed, in WT control mice, although occurring earlier in the former. Nonetheless, microscopy analysis revealed markedly greater neurovascular damage in HSHA mice. Consistent with the hypothesis of increased neurovascular insult as a consequence of hepatic synthesis of TRL-Aβ, PET findings clearly demonstrated an age-associated increase in PiB uptake. We suggest that this was indicative of the marked abundance of amyloid-rich lipofuscin aggregates distributed within brain parenchyma and within vessel lumen.

In this study, the assessment of cognitive function in HSHA mice was limited to an established sensitive fear-motivated challenge of hippocampal-dependent function, and at 12 months, showed significant treatment differences, with HSHA performing more poorly. In future studies, a more rigorous evaluation of behaviour as described by Cao and colleagues would be informative in considering the HSHA strain in the context of AD-related cognitive deficits [38]. Notably, the findings in this study demonstrate a level of compromised cognitive performance decline that is comparable to more amyloidogenic-aggressive and CNS-Aβ dominant transgenic strain of mice. Studies by Liu and colleagues showed that latency time for APP/PS1 mice in response to foot shock protocol as described here, mice at 12 months of age performed substantially worse compared to age-matched control mice [39].

The findings of this study and in earlier studies in WT mice on SFA-enriched diets suggest that chronically exaggerated vascular exposure to TRL-Aβ accelerates age-associated breakdown of cerebral capillaries and the NVU and that this occurs prior to late-stage amyloidogenic pathology being realised. Evidence that the metabolism of TRL-Aβ is a significant risk factor for AD provides new opportunities to consider prevention prospects through diet, lifestyle, and, indeed, the arsenal of lipid modulating drugs that are available to treat dyslipidemia and, by extension, possibly Aβ metabolism. It is worth noting that the HSHA strain studied was normolipidemic and comparable to age-matched WT controls, consistent with the hypothesis that exposure to lipoprotein-Aβ and not lipids per se is the likely primary trigger for NVU disruption. In one clinical study, there was an interesting observation that while patients with AD were otherwise normolipidemic, when challenged with a relatively small fat challenge of approximately 40 g (with 25 g of SFA), a remarkably amplified response of postprandial lipoproteins (CMs) compared to age-matched healthy controls was suggested [5]. Clinical studies suggest that patients with AD may experience exaggerated transient exposure to dietary induced TRL-Aβ, which preclinical models demonstrate compromise NVU integrity.

This study demonstrates a lipoprotein-Aβ axis for capillary disruption and, thereafter, nonspecific parenchymal extravasation of the lipoprotein-Aβ moiety in HSHA mice. The findings are consistent with the proposition of Sutcliffe and colleagues [40] suggesting liver, not brain, as the origin of brain Aβ deposits. Sutcliffe showed that reducing presenilin-2 by administration of a cancer therapeutic that does not cross the blood–brain barrier reduced accumulation of Aβ in both blood and brain. However, the authors acknowledge that in addition to a lipoprotein-Aβ capillary axis for CNS amyloid abundance, other transport processes can occur at the endothelial plasma membrane. The receptor for advanced glycation end products and the low-density lipoprotein receptor-related protein-1 can regulate the influx and efflux of soluble Aβ, respectively [41–45]. With respect to the latter, Sagare and colleagues showed that infusion with low-density lipoprotein receptor-related protein (LRP) cluster IV decreased brain abundance of Aβ concomitant with an increase plasma abundance [46]. The findings of Sagare and colleagues suggest that peripheral soluble LRP can serve as a peripheral sink for blood Aβ that may be relevant to the metabolism of TRL-Aβ in blood and brain [46].

In the APP KM670/671NL model with ubiquitous tissue expression, Richards and colleagues reported that amyloid plaques appeared by 17 to 18 months in the neocortex and hippocampus [47]. The HSHA mice were modelled on the APPKM670/671NL (Swedish) mutations, but with expression restricted to liver. In HSHA mice, the plasma abundance of Aβ was comparable to measures in humans (approximately 170 pg/mL for Aβ1–40 and 35 pg/mL for Aβ1–42), but substantially less than commonly used transgenic amyloid mice. For example, APP/PS1 mice have approximately at 171 pg/mL for Aβ1–42 and 480 pg/mL for Aβ1–40 [8]. In this study, potentially immature proteinaceous deposits were seen rarely compared to the relatively significant plaque formation in the APP/PS1 mice to 18 months of age (S7A Fig). Spectral analysis of potential proteinaceous deposits suggested a propensity for β-sheet formation (S7B Fig). While HSHA mice need to be investigated to older age than 18 months, the findings presented in this study nonetheless support a now large body of evidence that demonstrates that the genesis of plaque is not the initiating trigger for neurodegenerative processes to be initiated, but rather, may be consequential. This study provides evidence that more subtle chronic interactive effects of peripheral metabolism of TRL-Aβ with the cerebrovasculature may be sufficient to potentially cause AD.

In the context of translatability, a comprehensive clinical understanding of the peripheral metabolism of lipoprotein-Aβ would be highly informative in understanding risk for AD. Patients with a propensity for exaggerated biosynthesis of TRL-lipoproteins, or reduced clearance of TRL; putative synergistic effects with dietary fats, or with apo E genotype, will be critical for considering potential AD risk reduction strategies. The latter may include lifestyle and particularly dietary interventions for prevention, or reconsideration of lipid-modulating drugs in positively modulating lipoprotein-Aβ metabolism. Indeed, a clinical trial exploring the putative efficacy of probucol on cognitive performance in patients with mild cognitive impairment/early AD has been proposed based on the findings of potent suppression of Aβ biosynthesis and lipoprotein-associated secretion in preclinical models [35,37].

## Materials and methods

### Study design

This study aimed to investigate the involvement of the TRL-Aβ lipoprotein metabolic cascade and the risk of developing AD. To accomplish this, we developed a new model with humanised *APP* transgenes restricted exclusively to hepatocytes (HSHA strain), in order to investigate the peripheral metabolism of human amyloid hypothesis, in absence on CNS overexpression of amyloid. The effects of hepatic-specific humanised APP on memory performance, lipid/lipoprotein abundance in the brain, neurodegenerative changes, changes in regional brain volumes, indices of cerebral capillary function and neuroinflammation, and in vivo cerebral amyloid uptake were assessed in HSHA mice and their age-matched controls randomised to either 4, 8, 12, 18, or 24 months of age (*n =* 3 for in vivo PET and MRI imaging; *n* = 5 to 12 for biochemical analyses). Sample sizes were adequately powered to observe possible effects based on preliminary studies and past studies. The memory tests were conducted blinded to age and genotype by experienced investigators. All data collection and quantitative measures were performed by investigators blinded to sample identities until unblinding for final interpretation of statistical results.

### Generation of transgenic mice

Generation of a transgenic mouse model of hepatocyte-specific human amyloid (HSHA) was achieved via targeted gene knock-in technology by Ozgene (W.A, Australia). A targeting construct consisting of human APP695 cDNA containing the Swedish (KM670/671NL) and Indiana (V717F) mutations separated from the human ubiquitin c promoter by a floxed STOP cassette was targeted into the ROSA26 locus of C57BL/6J Bruce4 embryonic stem (ES) cells via homologous recombination [48]. Targeted ES cells were injected into goGermline blastocysts [49]. Male chimeric mice were obtained and crossed to C57BL/6J females to establish heterozygous germline offspring, which were subsequently bred to homozygosity, on a C57BL/6J background. The initial gene activation was achieved via cre-mediated deletion of the stop cassette by crossing to the liver-specific cre line B6.Cg-*Speer6-*^*ps1Tg(Alb-cre)21Mgn*^/J (Jax stock No. 003574) [50]. The Alb-Cre line we selected is widely used and thoroughly characterised. Originally created by Postic and colleagues [51], the line was characterised when generated by crossing to a lacZ expression reporter line, with expression shown by Postic and others’ laboratories (Gu, Stiles, and Lee) as being specific to the liver, with no expression observed in pancreas, spleen, kidney, skeletal muscle, or brain [52–54]. Cre recombination efficiency was found to be 40% efficient at birth, followed by 60% at 1 week old and 75% efficiency by 3 weeks. Additionally, Gu found that there was no recombination observed at gestational day 14, indicating that Cre expression commences close to term.

All animal procedures including the generation of the new transgenic line were approved by the National Health Medical Research Council–accredited Curtin Animal Ethics Committee (approval no. 2016–25) according to Australian code for the care and use of animals for scientific purposes.

### Analysis of human APP mRNA expression in HSHA mice

Liver, brain, lung, and duodenum samples were harvested, snap frozen in liquid nitrogen, and stored at −80°C. mRNA was extracted from frozen tissue using PureLink RNA Mini Kit (Thermo Fisher Scientific) as per supplied protocols. DNAse digestion on column was included as a control. Extracted mRNA was reverse transcribed using High-Capacity cDNA Reverse Transcription kit (Thermo Fisher Scientific) as per supplied protocol. Reverse transcription controls without enzyme were included, and RNA passed quality controls. The qPCR assay was designed to detect human APP with a high degree of specificity, and assays confirm that there was no detection of murine APP. Detection of qPCR signal demonstrates the expression of human APP in mRNA. qPCR of RT-cDNA was performed in duplex with eukaryote translation elongation factor 2 (Eef2, MGI: 95288) as endogenous control.

### Animal maintenance and sample collection

Male HSHA mice were maintained on standard maintenance chow (AIN93M, Specialty Feeds, W.A, Australia). At the age of 4, 6, 8, 12, and 18 months, the mice were killed through cardiac puncture under isoflurane anaesthesia. Brain tissue was collected into PBS, and a sagittal cut was made. The left hemisphere was immediately snap frozen in liquid nitrogen. The right hemisphere was fixed in 4% paraformaldehyde for 24 hours and cryoprotected in 20% sucrose for 72 hours before freezing in dry ice/isopentane. All plasma and tissue samples were stored at −80°C until next use.

### Sudan IV lipid staining

Approximately 20-μm thick fixed frozen brain sections were air dried and fixed in 4% paraformaldehyde for 5 minutes. Following a rinse in 50% ethanol, the sections were incubated in 1% Sudan IV solution in 50:50 acetone:70% ethanol (Alfa Aesar, MA, US) for 15 minutes. The sections were differentiated in 40% ethanol for 30 seconds, and the nuclei were stained with haematoxylin. The slide was then mounted in an aqueous mounting medium. The bright field images of the entire HPF were captured with Olympus BX-51 microscope at 10X objective. The number and size of lipid droplet staining were analysed with Zeiss Zen Blue v2.6 Image Processing Module. The lipid droplets were identified by applying a threshold-based binary mask.

### Direct spectroscopic lipid imaging

FTIR was used to analyse the relative abundance of lipids within the hippocampus. FTIR spectroscopic images were collected from the hippocampus and surrounding corpus callosum as detailed previously [55]. Briefly, FTIR images were acquired from 10-μm thick coronal brain sections, which were mounted onto 1-mm thick infrared transparent CaF_2_ substrate (Crystran). FTIR spectroscopic images were captured at 4 cm^−1^ spectral resolution with 16 coadded scans, with a Nicolet iN 10MX microscope fitted with a 8 × 2 pixel liquid nitrogen cooled linear array detector (25 μm/pixel). Background spectra were acquired under the same conditions from a blank region of the CaF_2_ substrate.

Analysis of FTIR data was performed using Cytospec v2.00.03 and OPUS v7.0 software. FTIR false colour images were generated for lipid ester distribution using the integrated area under the curve (iAUC) from 1,755 to 1,715 cm^−1^, as previously described [43,56,57]. Lipid images were analysed in ImageJ to determine the proportion of the hippocampus containing elevated lipid content, defined as iAUC value >0.6.

### Assessment of blood–brain barrier integrity

The integrity of the blood–brain barrier was assessed by quantitatively determining the perivascular extravasation of plasma-borne IgG, as established previously [58]. Briefly, 20-μm thick coronal brain sections from fixed frozen right hemispheres were blocked with 10% goat serum for 30 minutes and incubated with goat anti-mouse IgG conjugated with Alexa488 (1:100, Thermo Fisher Scientific) for 20 hours at 4°C. Following nuclear staining with DAPI, the sections were mounted and observed with UltraVIEW Vox confocal microscopy. Confocal 3D images consisting of 20 z-stack images were captured with 20X objective. Approximately twenty 3D images were randomly taken by from each CTX and hippocampal region to cover the majority of the area in each region. Perivascular distribution of IgG was detected by using machine learning segmentation of the cerebral capillary vessels, as described previously [58,59]. The sum voxel intensity of the IgG fluorescent dye was calculated and expressed as per image (volume unit).

The expression of a blood–brain barrier tight junction, occludin-1, was also determined with quantitative immunofluorescent microscopy. Briefly, the 20-μm thick brain cryosections were incubated with anti-occludin (1:500, Thermo Fisher Scientific). Subsequently, the sections were incubated with anti-rabbit Alexa 546 (1:500, Thermo Fisher Scientific). The fluorescent images were captured with Zeiss Axioscan Z.1., and the pixel intensity of occludin-1 staining was analysed with Zeiss Zen Blue.

Vascular density was also measured by using laminin-a4 staining of the cerebrovasculature. The brain cryosections were incubated with anti-laminin a4 antibody (1:200, R&D Systems) followed by anti-rat Alexa488 (1:100, Thermo Fisher Scientific). The fluorescent images were captured with Zeiss Axioscan Z.1., and the pixel intensity of vessel area was analysed with Zeiss Zen Blue.

### Glial and astrocyte activation

As a marker of neuronal inflammation, microglial activation, astrocyte activation, and astrocytosis were determined by using ionised calcium-binding adaptor molecule 1 (Iba-1), complement component 3 (C3), and GFAP, respectively. For Iba-1 staining, 20 μm cryosections prepared from fixed frozen right hemispheres were incubated in 10 mM T.E. buffer (pH 6.0) for 20 hours at 37°C for antigen retrieval. The sections were then incubated with a primary antibody of anti-Iba-1 (1:500, Novachem), anti-C3 (1:100, Abcam (Cambridge, United Kingdom)), or anti-GFAP (1:200, Abcam) for 20 hours at 4°C. Subsequently, Iba-1 and GFAP were detected by using anti-rabbit IgG conjugated with Alexa488, while C3 was detected with anti-rat IgG Alexa488 for 2 hours at 20°C. Confocal images were randomly captured with UltraVIEW Vox with 20X objective by a blinded investigator. Zeiss ZEN Intellisis trainable segmentation module was used to identify the stained astrocytes and microglia. The intensity of the staining was calculated per image.

### Analyses of neurodegeneration

Degenerating neurons were detected with Fluoro-Jade C staining kit (Biosensis) according to the manufacturer’s instruction [60]. Briefly, 20 μm fixed frozen sections were incubated in sodium hydroxide (Solution A) for 5 minutes and then in potassium permanganate (Solution B) for 10 minutes. Finally, the sections were incubated in Fluoro-Jade solution (Solution C) with DAPI (Solution D) for 10 minutes in dark conditions. Confocal 3D images were captured with UltraVIEW Vox with 20X objective. In order to cover the majority of each region area, approximately 20 images were randomly taken from the CTX and HPF by a trained investigator. The number of positively stained neurons was manually counted by a blinded investigator.

### Assessment of cell apoptosis

The apoptotic cells were detected with commercial TUNEL assay kit according to the manufacturer’s instruction (Abcam). Briefly, 20-μm thick cryosections were incubated with terminal deoxynucleotidyl transferase. Biotinylated nucleotides were detected with a streptavidin-horseradish perisidase. Diaminobenzidine was used to detect the TUNEL positive cells, with brown colour. Following the staining, bright field microscopy images were captured with Zeiss AxioScan Z.1. with Hitachi HV-F2032SCL camera. Zeiss Zen Blue 3.1 Intellesis segmentation was used to identify total number of nuclei based on its colour and shape. Subsequently, the segmentation was done based on its colour to identify TUNEL positive (brown: green <180 and blue <150) and negative cells. The data are presented as TUNEL positive cell number per total cell number.

### Measurement of brain regional volume

Three-dimensional volumes of brain CTX, hippocampus, and combined lateral, third, fourth, and cerebral aqueduct ventricles were measured with MRI.

### Animal preparation for MRI scans

Mice were anaesthetized with isofluroane (2%), and eyes were protected with Lacri-Lube gel. The head was fixed using a brain coil. Respiration and heart rate were monitored throughout the entire scan. The total imaging time was approximately 30 minutes per animal. MRI scans were performed on HSHA mice and their age-matched controls at 8, 12, and 18 months of age (*n =* 2 to 3).

### MRI acquisition protocols

T2-weighted MRI scans were acquired for 18 mice with a 3T micro-MRI Scanner (MR Solutions, UK). A total of 12 coronal, axial, and sagittal sections were obtained using conventional Fast Spin Echo (FSE) T2-weighted sequence (0.8 mm slice thickness, 256 × 256 matrix).

Images were reconstructed, processed, and analysed using Vivoquant Software Version 4.0 (inviCRO, Boston, MA). For volumetric analysis, MRI scans in the coronal plane were segmented for quantification using VivoQuant. Regions of interest were manually defined in VivoQuant based on the Allen Mouse Brain Atlas [61] comprising the cerebral CTX, HPF, and total ventricular volume as a sum of the lateral ventricles, third ventricle, fourth ventricle, and aqueduct. A blinded investigator was assigned this task.

### Detection of cerebral amyloidosis with in vivo PET/CT

A subset of mice at 6, 12, and 18 months of age were analysed with ^11^C-PiB PET/CT in vivo imaging to quantitatively assess the burden of cerebral amyloidosis.

### Animal preparation for PET/CT scans

Mice were injected intravenously with approximately 20 MBq of ^11^C PiB (Department of Medical Technology and Physics, QEII, Sir Charles Gairdner Hospital) through tail vein and placed in a lead lined box for an uptake period of 20 minutes. Following uptake, each animal was anaesthetized with gaseous isofluorane (2%), and eyes were protected with Lacri-Lube gel and placed into the PET scanner bed in a supine position and secured with tape. Respiration was monitored throughout the entire scan. The total imaging time was approximately 20 minutes per animal and 10 minutes for computed tomography (CT).

### PET/CT acquisition protocols

HSHA mice and their age-matched controls at 6, 12, and 18 months of age (*n =* 3/group) were imaged using a small animal microPET/CT scanner (Sedecal nanoPET/CT, Sedecal, Spain). In vivo PET scans were obtained immediately after the uptake period. A 20-minute static scan of the brain was acquired with a 100- to 700-KeV energy window. Acquired data reconstructed with 3D-OSEM iterative reconstruction using 3 iterations 16 subsets, with scatter and random correction. Low-dose CT was performed for attenuation correction and anatomical localization. The reconstructed PET/CT data were processed and analysed using Vivoquant Software Version 4.0 (inviCRO, Boston, MA). The PET data were fused with the MRI using the low-dose CT for anatomical correction.

### Quantitation of cerebral PiB uptake

To quantify cerebral PiB uptake in particular subregions of interest, reconstructed CT images were directly coregistered to a T1-weighted MRI-based template from the Australian Mouse Brain Mapping Consortium (AMBMC) [62]. To achieve this intermodality coregistration, each CT image was cropped to include only the skull and converted to a binary mask. Thereafter, a skull cavity mask was created and used as a coregistration target for the T1 template; which is achieved via an affine transformation using FSL software [63]. Thereafter, volumes of interest, including whole brain, CTX, hippocampus, and cerebellum for each mouse, were applied to their corresponding reconstructed PET images to calculate the ^11^C PiB whole brain-to-cerebellum (SUVR_WB:CBL_) SUVRs.

### Passive avoidance assessment

The passive avoidance apparatus was divided into light and dark compartments (38 × 9 × 17 cm) separated by a sliding door (Ugo Basile, Comerio, Italy). Comprising of a 2-day protocol, each mouse was placed in the light compartment for 30 seconds on the initial training/acquisition trial. After 30 seconds, the door separating both compartments opened. Once the mouse enters the dark compartment, the door closed immediately and an electrical foot shock (0.1 mA) was delivered via the grid floor for 0.1 seconds by a stimulator. The mouse was then returned to its home cage. Approximately 24 hours post-training, each mouse was subjected to the retention trial where they were once again placed in the illuminated chamber for 30 seconds followed by opening of the trap door after 30 seconds. The latency time was defined as the time it took a mouse to enter the dark chamber with a maximum of 300 seconds.

### Transmission electron microscopy

Brain hippocampal or cortical tissues were cut into 1 mm cubes and placed in 2.5% glutaraldehyde (Merck, NJ, US) in 0.1 M cacodylate buffer (pH 7.4) (Fluka) and postfixed for 2 hours in 0.5% osmium tetroxide (Polysciences) in 0.1 M cacodylate buffer. Tissues were rinsed in 0.08 M phosphate buffer. To enable better membrane contrast, a solution of 1% OsO_4_ and 1% potassium ferrocyanide (Merck) in 0.1 M sodium cacodylate buffer (pH 7.4) was used during processing. Tissues were then dehydrated in ascending concentrations of ethanol, vacuum embedded in Epon-Araldite (overnight), and placed in a 60°C oven overnight to cure. During all procedures, tissues were continuously agitated to ensure even infiltration of solutions into the tissue. The tissue block was then trimmed, and ultrathin sections of a pale silver interference colour (approximately 100 nm) were cut using a Diatome diamond knife (Leica, Perth, Australia) on an LKB Nova ultratome and picked up onto uncoated 200-mesh copper grids (Maxtaform HF33Cu, Taab Laboratories, UK). TEM imaging was carried out on a JEOL 2100 TEM with a LaB6 source operating at 120 kV and equipped with a Gatan Orius SC100 11Mpix CCD camera. The TEM analyses were conducted by a blinded investigator.

### Statistical analyses

All data are expressed as mean ± SEM. The residuals of the robust fit were analysed for each data set to identify any potential outliers. This step uses an outlier test adapted from the false discovery rate approach of testing for multiple comparisons. On cleaned data with outliers removed, an unpaired *t* test with Welch correction testing for nonequivalence of standard deviations was utilised. The effects of age and strain on brain hippocampal lipid accumulation were analysed by using two-way ANOVA (mouse strain and age were independent factors) followed by post hoc testing of multiple comparisons (*t* test). The statistical significance of multiple comparisons was assessed at *p* < 0.05. Memory retention measures that were not normally distributed were analysed by nonparametric Krusal–Wallis test, followed by Dunn multiple comparisons test. Pearson correlation coefficient analysis was used to assess statistical associations.

### Plasma apo B and amyloid concentrations

Plasma and brain concentrations of apo B was measured with commercial ELISA kit (Abcam ab230932) according to manufacturer’s instruction. Mouse Aβ concentrations were determined with commercial ELISA (Wako 294–62501 and 292–64501), while human Aβ was measured with Simoa Human Neurology 3-plex A (N3PA) according to manufacturer’s instruction.

## Supporting information

S1 Data Excel spreadsheet containing, in separate sheets, the numerical data for Figs 1A, 1B, 2A–2F, 3A, 3B, 3Y, 4B–4G, 5A–5H and 7, Tables 1 and 2, and S1–S5. (XLSX)

S1 Fig Weights. The mean weight of HSHA and WT control mice is presented. Two-way ANOVA with Fisher LSD multiple comparison was used to assess the significance (** *p* < 0.01, *** *p* < 0.001). The data underlying S1 Fig can be found in S1 Data. HSHA, hepatocyte-specific human amyloid; WT, wild-type.
(TIFF)

S2 Fig TUNEL assay for cell apoptosis. The rate of cell apoptosis was determined in HSHA mice in comparison to WT control mice by using a commercial TUNEL assay kit. The TUNEL positive and negative cells were identified based on its colour with automated segmentation of Zeiss Zen image analysis software. (A) The number of apoptotic cells is presented per total cell number. Statistical significance was assessed by two-way ANOVA, and individual *p*-values are presented in the graph. (B) Representative microscopy images are shown from WT control and HSHA mice with corresponding auto-segmentation results (blue: negative and red: positive). The data underlying S2 Fig can be found in S1 Data. HSHA, hepatocyte-specific human amyloid; WT, wild-type.
(TIFF)

S3 Fig Quantitative immunomicroscopy analysis of cerebral capillary occludin-1 expression. (A) The expression of blood–brain barrier tight junction protein, occludin-1, was quantitatively assessed by immunofluorescent microscopy in HSHA mice and age-matched WT control mice. The expression is expressed as pixel intensity per vessel area. Two-way ANOVA followed by Fisher LSD multiple comparison was used and indicated with ** at *p* < 0.01. (B) Representative immunomicrographs of occludin-1 (green) and IgG (magenta) show colocalization of loss of tight junctions and IgG extravasation. The data underlying S3 Fig can be found in S1 Data. CTX, cortex; HPF, hippocampal formation; HSHA, hepatocyte-specific human amyloid; IgG, immunoglobulin G; WT, wild-type.
(TIFF)

S4 Fig Vascular density. The density of cerebrovasculature was quantitatively assessed in the CTX and hippocampal regions of HSHA mice, in comparison to age-matched WT mice. The data are presented as vascular area (detected with laminin-a4 staining) per image. Two-way ANOVA was used to assess the statistical significance (no significance detected). The data underlying S4 Fig can be found in S1 Data. CTX, cortex; HPF, hippocampal formation; HSHA, hepatocyte-specific human amyloid; WT, wild-type.
(TIFF)

S5 Fig Assessment of liver function. The effects of conditional human APP gene knock-in on liver function was tested in HSHA and WT control mice at 6, 12, and 18 months of age by using ALT assay and histological examination. (A) Plasma levels of ALT activity was tested with a commercial assay kit and presented as relative to control. Two-way ANOVA was used to assess the statistical significance (* *p* < 0.05). (B) A representative H&E histological image is presented in 12-month-old HSHA mice, showing moderate sign of steatosis. No other histopathological changes were observed. The data underlying S5 Fig can be found in S1 Data. ALT, alanine aminotransferase; APP, amyloid precursor protein; H&E, hematoxylin and eosin; HSHA, hepatocyte-specific human amyloid; WT, wild-type.
(TIFF)

S6 Fig Ventricular lipid droplets are present in aged HSHA mice. **(A).** Representative bright field micrograph showing Sudan IV staining along the lateral ventricular wall of the brain of a 12-month-old HSHA mouse; scale bar depicts 50 μM. **(B, C)** Magnified micrographs of the indicated areas indicated by the white rectangles in (A); scale bar = 10 μM. HSHA, hepatocyte-specific human amyloid.
(TIFF)

S7 Fig FTIR spectroscopic imaging of individual Aβ-plaques in HSHA and APP/PS1 mice. Representative FTIR images showing the distribution of aggregated protein (red) and lipid ester (blue) in the HPF of a **(A)** 6-month-old HSHA mouse (left) and 9-month-old APP/PS1 amyloid transgenic positive control mouse with ubiquitous amyloid expression in CNS (right); (B) propensity of 4-month-old (blue) and 8-month-old (red) HSHA mice to form β-sheet aggregates versus mature APP/PS1 amyloid transgenic mice (dotted) and WT control mice (black). Aß, amyloid beta; CNS, central nervous system; FTIR, Fourier transform infrared; HPF, hippocampal formation; HSHA, hepatocyte-specific human amyloid; WT, wild-type.
(TIFF)

## Abbreviations

Aß: amyloid beta
AD: Alzheimer disease
ALT: alanine aminotransferase
AMBMC: Australian Mouse Brain Mapping Consortium
apo B: apolipoprotein B
APP: amyloid precursor protein
CNS: central nervous system
CT: computed tomography
CTX: cortex
C3: complement component 3
Eef2: eukaryote translation elongation factor 2
ES: embryonic stem
FSE: Fast Spin Echo
FTIR: Fourier transform infrared
GFAP: glial fibrillary acidic protein
HPF: hippocampal formation
HSHA: hepatocyte-specific human amyloid
iAUC: integrated area under the curve
Iba-1: ionised calcium-binding adaptor molecule 1
IgG: immunoglobulin G
IHC: immunohistochemical
LIB: lipid inclusion body
LRP: lipoprotein receptor-related protein
MRI: magnetic resonance imaging
NVU: neurovascular unit
N3PA: Neurology 3-plex A
PET: positron emission tomography
PiB: Pittsburgh compound
PS: presenilin
SFA: saturated fatty acid
SUVR: standardised uptake value ratio
TEM: transmission electron microscopy
TRL: triglyceride-rich lipoprotein
VLDL: very low-density lipoprotein
WT: wild-type
