# Supplementary figures and images for: Synthesis of human amyloid restricted to liver results in an Alzheimer disease–like neurodegenerative phenotype
Source: PLoS Biol. 2021 Sep 14;19(9):e3001358. doi: 10.1371/journal.pbio.3001358 (PMC8439475; doi:10.1371/journal.pbio.3001358)

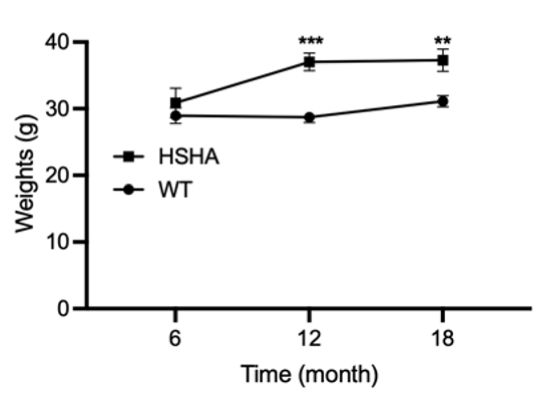

Supplement: S1 Fig — The mean weight of HSHA and WT control mice is presented. Two-way ANOVA with Fisher LSD multiple comparison was used to assess the significance (** p < 0.01, *** p < 0.001). The data underlying S1 Fig can be found in S1 Data. HSHA, hepatocyte-specific human amyloid; WT, wild-type. (TIFF) [file pbio.3001358.s002.tiff]

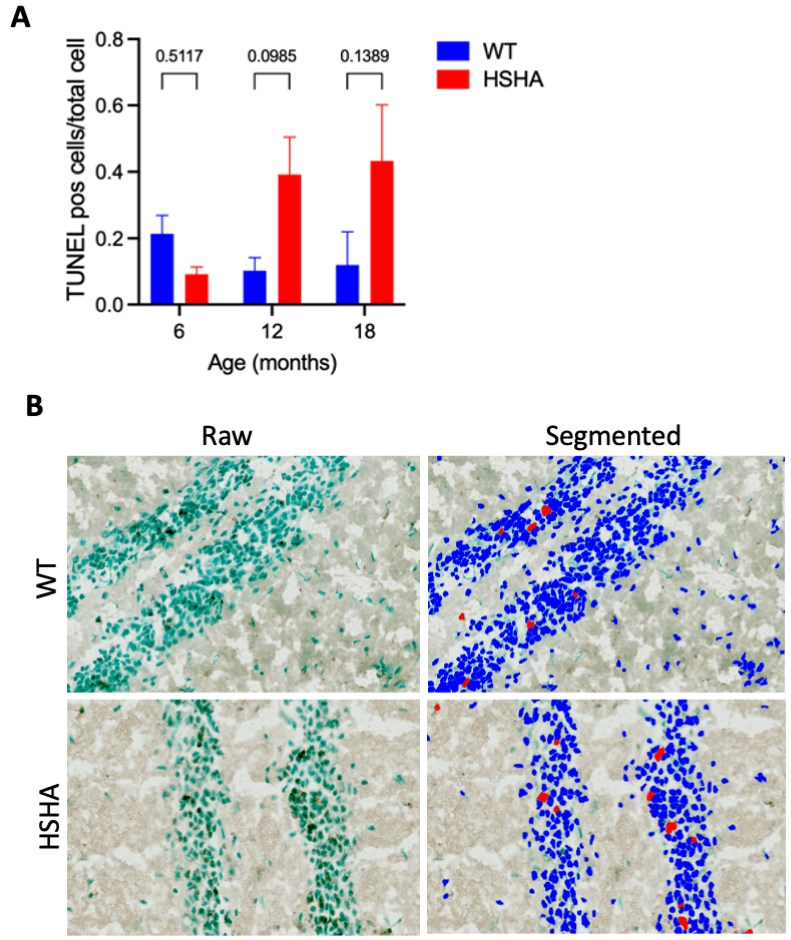

Supplement: S2 Fig — The rate of cell apoptosis was determined in HSHA mice in comparison to WT control mice by using a commercial TUNEL assay kit. The TUNEL positive and negative cells were identified based on its colour with automated segmentation of Zeiss Zen image analysis software. (A) The number of apoptotic cells is presented per total cell number. Statistical significance was assessed by two-way ANOVA, and individual p-values are presented in the graph. (B) Representative microscopy images are shown from WT control and HSHA mice with corresponding auto-segmentation results (blue: negative and red: positive). The data underlying S2 Fig can be found in S1 Data. HSHA, hepatocyte-specific human amyloid; WT, wild-type. (TIFF) [file pbio.3001358.s003.tiff]

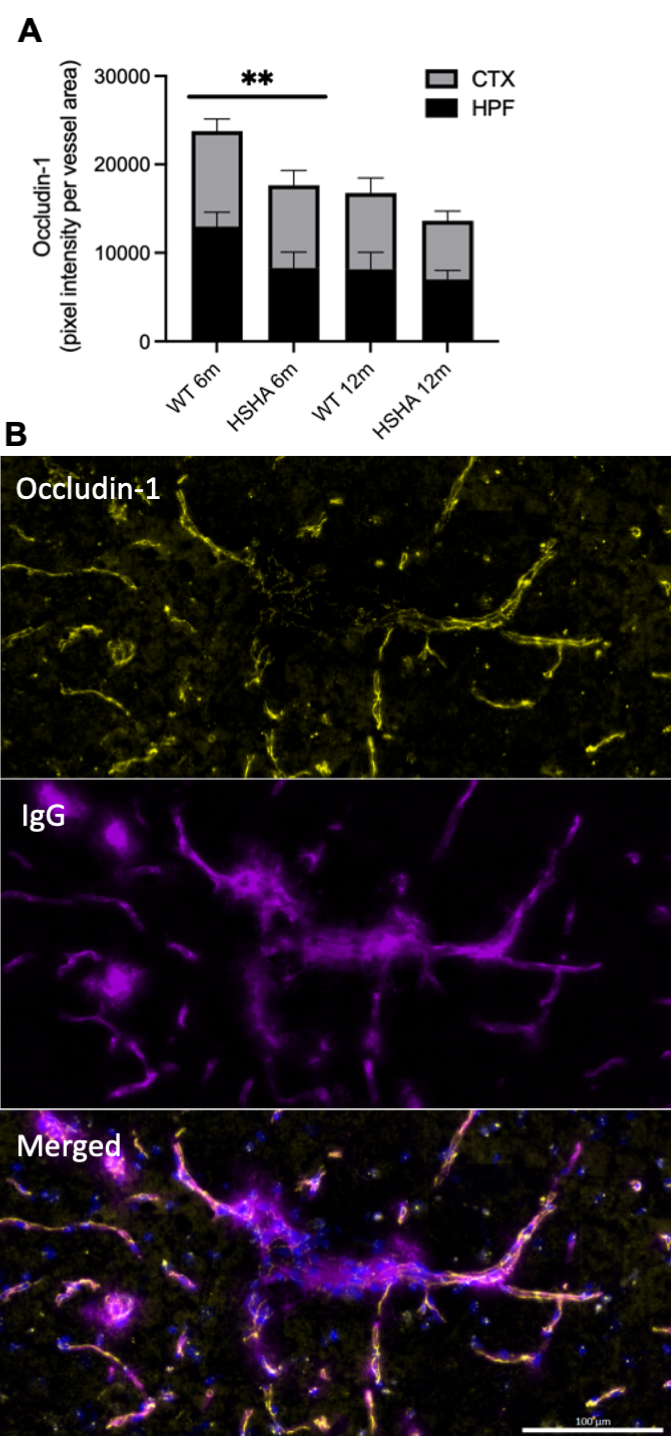

Supplement: S3 Fig — (A) The expression of blood–brain barrier tight junction protein, occludin-1, was quantitatively assessed by immunofluorescent microscopy in HSHA mice and age-matched WT control mice. The expression is expressed as pixel intensity per vessel area. Two-way ANOVA followed by Fisher LSD multiple comparison was used and indicated with ** at p < 0.01. (B) Representative immunomicrographs of occludin-1 (green) and IgG (magenta) show colocalization of loss of tight junctions and IgG extravasation. The data underlying S3 Fig can be found in S1 Data. CTX, cortex; HPF, hippocampal formation; HSHA, hepatocyte-specific human amyloid; IgG, immunoglobulin G; WT, wild-type. (TIFF) [file pbio.3001358.s004.tiff]

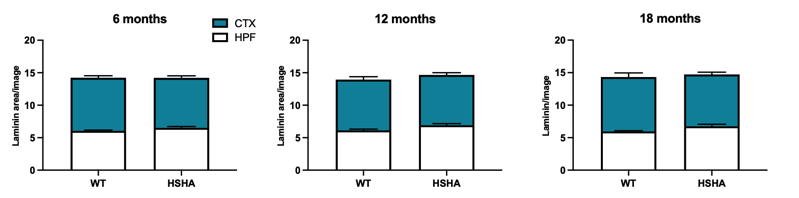

Supplement: S4 Fig — The density of cerebrovasculature was quantitatively assessed in the CTX and hippocampal regions of HSHA mice, in comparison to age-matched WT mice. The data are presented as vascular area (detected with laminin-a4 staining) per image. Two-way ANOVA was used to assess the statistical significance (no significance detected). The data underlying S4 Fig can be found in S1 Data. CTX, cortex; HPF, hippocampal formation; HSHA, hepatocyte-specific human amyloid; WT, wild-type. (TIFF) [file pbio.3001358.s005.tiff]

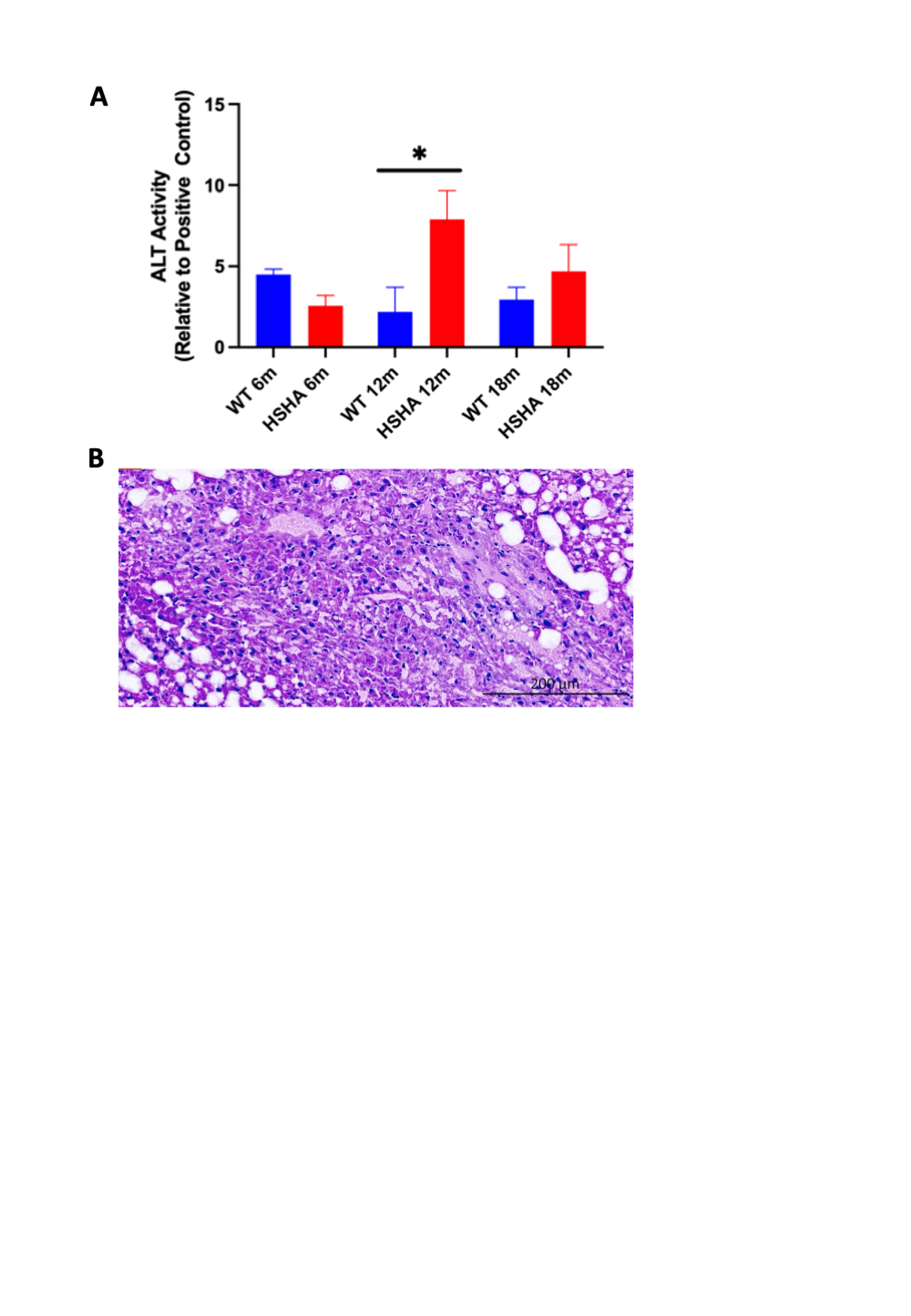

Supplement: S5 Fig — The effects of conditional human APP gene knock-in on liver function was tested in HSHA and WT control mice at 6, 12, and 18 months of age by using ALT assay and histological examination. (A) Plasma levels of ALT activity was tested with a commercial assay kit and presented as relative to control. Two-way ANOVA was used to assess the statistical significance (* p < 0.05). (B) A representative H&E histological image is presented in 12-month-old HSHA mice, showing moderate sign of steatosis. No other histopathological changes were observed. The data underlying S5 Fig can be found in S1 Data. ALT, alanine aminotransferase; APP, amyloid precursor protein; H&E, hematoxylin and eosin; HSHA, hepatocyte-specific human amyloid; WT, wild-type. (TIFF) [file pbio.3001358.s006.tiff]

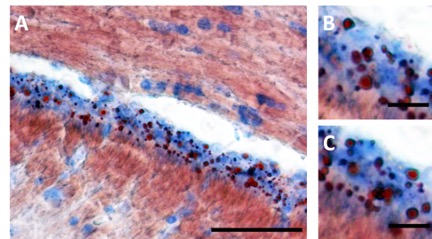

Supplement: S6 Fig — (A). Representative bright field micrograph showing Sudan IV staining along the lateral ventricular wall of the brain of a 12-month-old HSHA mouse; scale bar depicts 50 μM. (B, C) Magnified micrographs of the indicated areas indicated by the white rectangles in (A); scale bar = 10 μM. HSHA, hepatocyte-specific human amyloid. (TIFF) [file pbio.3001358.s007.tiff]

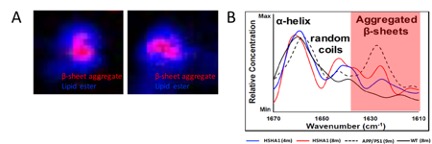

Supplement: S7 Fig — Representative FTIR images showing the distribution of aggregated protein (red) and lipid ester (blue) in the HPF of a (A) 6-month-old HSHA mouse (left) and 9-month-old APP/PS1 amyloid transgenic positive control mouse with ubiquitous amyloid expression in CNS (right); (B) propensity of 4-month-old (blue) and 8-month-old (red) HSHA mice to form β-sheet aggregates versus mature APP/PS1 amyloid transgenic mice (dotted) and WT control mice (black). Aß, amyloid beta; CNS, central nervous system; FTIR, Fourier transform infrared; HPF, hippocampal formation; HSHA, hepatocyte-specific human amyloid; WT, wild-type. (TIFF) [file pbio.3001358.s008.tiff]
